# A noninvasive flexible conformal sensor for accurate real-time monitoring of local cerebral edema based on electromagnetic induction

**DOI:** 10.7717/peerj.10079

**Published:** 2020-10-06

**Authors:** Jingbo Chen, Gen Li, Mingsheng Chen, Gui Jin, Shuanglin Zhao, Zelin Bai, Jun Yang, Huayou Liang, Jia Xu, Jian Sun, Mingxin Qin

**Affiliations:** 1 College of Biomedical Engineering, Third Military Medical University (Army Medical University), Chongqing, China; 2 School of Pharmacy and Bioengineering, Chongqing University of Technology, Chongqing, China; 3 China Aerodynamics Research and Development Center Low Speed Aerodynamic Institute, Mianyang, China; 4 Department of Neurosurgery, Southwest Hospital, Chongqing, China

**Keywords:** Cerebral edema, Magnetic Induction Phase Shift, Conformal two-coil structure, Flexible conformal mips sensor, Local focusing measurement, Bedside monitoring

## Abstract

Cerebral edema (CE) is a non-specific pathological swelling of the brain secondary to any type of neurological injury. The real-time monitoring of focal CE mostly found in early stage is of great significance to reduce mortality and disability. Magnetic Induction Phase Shift (MIPS) is expected to achieve non-invasive continuous monitoring of CE. However, most existing MIPS sensors are made of hard materials which makes it difficult to accurately retrieve CE information. In this article, we designed a conformal two-coil structure and a single-coil structure, and studied their sensitivity map using finite element method (FEM). After that, the conformal MIPS sensor that is preferable for local CE monitoring was fabricated by flexible printed circuit (FPC). Next, physical experiments were conducted to investigate its performance on different levels of simulated CE solution volume, measurement distance, and bending. Subsequently, 14 rabbits were chosen to establish CE model and another three rabbits were selected as controls. The 24-hour MIPS real-time monitoring experiments was carried out to verify that the feasibility. Results showed a gentler attenuation trend of the conformal two-coil structure, compared with the single-coil structure. In addition, the novel flexible conformal MIPS sensor has a characteristic of being robust to bending according to the physical experiments. The results of animal experiments showed that the sensor can be used for CE monitoring. It can be concluded that this flexible conformal MIPS sensor is desirable for local focusing measurement of CE and subsequent multidimensional information extraction for predicting model. Also, it enables a much more comfortable environment for long-time bedside monitoring.

## Introduction

Cerebral edema (CE), which can be defined as an accumulation of excessive fluid within either brain cells or extracellular spaces, is a non-specific pathological swelling of the brain secondary to any type of neurological injury like hemorrhage, ischemia, and traumatic brain injury (TBI) (Cook et al., 2020). CE is one of the highest risk factors for mortality and poor outcome, which occurs in more than 60% of patients with mass brain lesions and nearly 15% in those patients who showed no sign from their imaging diagnosis (Jha, Kochanek & Simard, 2019). The main physical effect of an uncontrolled increase of intracerebral volume is the rise of Intracranial Pressure (ICP), which causes insufficient cerebral blood flow (CBF), herniation of brain tissue and a subsequent brain death. CE complicates stroke and contributes to early neurological deterioration and poor outcome. A retrospective single-center study of 139 patients found that perihematomal edema (PHE) expansion rate in the first 24 h on admission was a significant predictor of 90-day mortality (Parry-Jones et al., 2015). Another study in 596 patients found that the increasing PHE expansion rate in the first 72 h after ICH is associated with higher mortality (Murthy et al., 2015). The change rate of CE growth is a new, emerging parameter and might represent a better indicator for poor outcome, compared with the commonly image-based diagnosis (Selim & Norton, 2020).

According to the Guidelines for the Early Management of Patients with Acute Ischemic Stroke (the American heart association/American Stroke association, AHA/ASA, 2018), there is still no fully acceptable method that can monitor and predict the progression of CE (Powers et al., 2018). Brain imaging methods, especially CT & MRI, are the gold standard to diagnose the development of CE (Olindo & Sibon, 2017). Routinely, CT scans (Dhar et al., 2018) can measure the progress of CE. However, those devices are heavy and fixed, which means they cannot carry out continuous bedside diagnosis. The progression of CE is difficult to retrieve timely by those traditional imaging modalities. The limitations of hospital medical resources also lead to patients not being able to repeatedly perform imaging examinations (Kalkonde et al., 2018). In practical terms, it is based on doctors’ experience whether to carry out imaging examination and further consultation. Thus, there is a certain blindness of imaging method considering individual differences between patients. Multi-modal Bedside monitoring which can be integrated into the ICU can serve as a supplement to imaging equipment and, in some cases, provide more guidance for medical staff (Kumar, Athar & Kamran, 2017). ICP is the most direct and widely used means of intracranial monitoring at present (Vanaclocha et al., 2017). Cerebral perfusion pressure (CPP), calculated by subtracting ICP from mean arterial pressure (MAP), reflects the pressure level of the blood reaching the brain, which represents the level of compensatory mechanisms. In fact, invasively and directly monitoring ICP can effectively reflect the severity of brain condition. However, that requires a probe being inserted into brain parenchyma, subarachnoid or dura matter, which bring secondary damage to patients. A portable, non-invasive method is needed (Rao et al., 2018).

Non-invasive monitoring methods have great potential advantages such as lower risk of infection and bleeding, mobility and safety (Vinciguerra & Bösel, 2017). Various non-invasive monitoring methods are gradually becoming new types of neurosurgical monitoring and have made many promising achievements. Transcranial Doppler (TCD) sonography can be used for cerebral monitoring via measuring cerebral blood flow in real time using high-frequency (2 MHz) acoustic waves (Rasulo et al., 2017). But continuous TCD is still under improvement. Electrical Impedance Tomography (EIT) has been proposed as a possible method to detect cerebral injuries (Song et al., 2018). Yet EIT requires current injection through a series of electrodes precisely placing on the corresponding position on the head, not mention that the current is “blocked” by the high resistivity skull which in turns may reduce the detection precision (Yan et al., 2017). Near Infrared Spectroscopy (NIRS) can detect brain injuries by using the near-infrared spectrum of light to penetrate brain tissue so as to estimate oxy/deoxy-hemoglobin concentration change (Yang et al., 2019b). But NIRS may fail to detect deep or early brain injuries due to its limited detection distance.

Electromagnetic induction theory provides a possible method for non-invasive bedside monitoring (Hopfer et al., 2017), considering the fact that skull does not block wide frequency range of electromagnetic waves compared to current and light. Monitoring the phase shift between excitation field and disturbing field generated by the induced current in brain tissue can theoretically reflect intracranial pathophysiological changes after CE (Griffiths, Stewart & Gough, 1999). González & Rubinsky (2006) built a simple mathematical model of electromagnetic induction in tissue and proved that it was possible to achieve non-contact detection of edema and hemorrhage in brain and other tissues. Subsequently, they carried out animal experiments and successfully proved that inductive bulk measurements of phase shift have the potential to detect changes in intraperitoneal fluid (González, Horowitz & Rubinsky, 2007). In 2013, they proposed that it is possible to successfully differentiate brain states through multi-frequency electromagnetic measurements and a Volumetric Electromagnetic Phase Shift Spectroscopy (VEPS) classifier-based technology (Gonzalez et al., 2013). Kellner et al. (2018) had applied a Volumetric Impedance Phase-shift Spectroscopy (VIPS) device (Cerebrotech Medical Systems, USA) and induced a bioimpedance asymmetry score to assess the severity of stroke. In 2020, Oziel et al. used a simple single coil device to detect changes due to fluid volume alteration in the brain, they had proved a sensitivity of 2 ml change in tissue/blood ratio (Oziel et al., 2020; Oziel, Korenstein & Rubinsky, 2020). Our research group has been focusing on the diagnosis of brain injuries via Magnetic Induction Phase Shift (MIPS) method. Jin et al. (2014) built a novel sensor structure and dramatically improved the sensitivity compared with the single excitation-receiving coil structure. In 2015, Pan et al. (2015) established a cerebral hemorrhage Magnetic Induction Phase Shift Spectroscopy (MIPSS) detection system and successfully distinguished the five states (pre-operation, post-operation, Blood injection 1 ml/2 ml/3 ml) of cerebral hemorrhage in rabbits. Sun et al. (2016) deduced the relationship between MIPS and ICP. In 2017, Li et al. built a real-time continuous CE monitoring system and the system is capable of monitoring the process of CE and reveal its severity (Li et al., 2017a; Li et al., 2017c). We also built a cerebral hemorrhage test system and proved that it can monitor the progressive development of cerebral hemorrhage in real-time (Li et al., 2017b). In 2019, we proved that our MIPS system can achieve the detection of early functional changes in Closed cerebral hemorrhage (CCH) as well as distinguish different severities of CCH, combined with CCH pathological mechanisms (Yang et al., 2019a).

Although the listed researches above provide sufficient evidence for the effective monitoring of cerebral edema by MIPS, it is difficult to accurately obtain CE information without considering the specific coil sensor design targeting specific subtypes and detection needs. The coil sensor is an important part of the MIPS detection system. Its structure and relative position to the lesion are the main factors affecting the measurement results. The studies on biological tissue Magnetic Induction Tomography (MIT) are based on the theory of the single-exciting single-induction coaxial coil. This structure is the most widely used to verify the effectiveness of the MIPS method. However, this structure is sensitive to those disturbance either near excitation coil or sensing coil (Rosell, Casanas & Scharfetter, 2001), which brings about a problem that it is hard to locate the lesion. Meanwhile, it cannot capture the exact physiological changes of edema in specific brain tissue or regions due to considering the brain as a whole measured object. More importantly, this structure needs hard materials to accurately fix the geometric relative position of the two coil sensors (Riedel et al., 2004; Xu et al., 2008) and it is unrealistic to move the patient’s head, especially critically ill patients, to the area with high sensitivity. For example, González et al. (2010) and Gonzalez et al. (2013) fabricated the excitation and sensing coils on a plastic harness specifically designed for human head to detect over-hydration. He et al. (2010) used plastic to fix sensors and put the sensor arrays into a closed and transparent container (He et al., 2010; Luo et al., 2012). From the perspective of clinical application, those sensors should be easy to wear and cause no difficulties to patients and medical staff (Qusba et al., 2014; Chen et al., 2017). Using printed circuit board (PCB) can reduce the use of hard materials (Zakaria et al., 2012), but PCB is still not suitable for direct clinical applications. Recently, Teichmann integrated a single-coil magnetic induction sensor into a shirt for cardiorespiratory activity monitoring using high frequency litz wire (Teichmann et al., 2013a; Teichmann et al., 2013b; Teichmann et al., 2014). But the sensitivity map of the single-coil sensor-which showed the sensitivity of the sensor to the measured object in different positions-was not discussed in those works. Hye Ran Koo built a wearable system for vital sign monitoring utilizing a textile-based inductive coil sensor (Koo et al., 2014; Sun et al., 2015). Results indicated that the inductive coil sensor is capable of heart rate monitoring and the position of the sensor significantly affected the quality of the measurement results. However, the sensitivity map of the single-coil sensor was unknown. As a derivative type of coaxial coil, conformal two-coil structure enables direct textile or flexible printed circuit (FPC) integration but its sensitivity map similarly remains to be studied. It is unclear which one is more suitable for MIPS-based CE detection and monitoring. Furthermore, the effect of the measurement-induced deformation after flexible fabrication on the results needs investigation.

In this article, we designed a conformal two-coil structure and a single-coil structure according to skull size of rabbits, and studied their sensitivity map using finite element method (FEM). Combined with the pathological features of local CE, we selected the conformal structure with high local focus performance and fabricated the coil sensor by FPC. Then, the physical experiments were conducted to investigate its performance on different levels of simulated cerebral edema solution volume, measurement distance, and bending. After that, the model of rabbit cerebral edema was established and the 24-hour MIPS real-time monitoring experiment was carried out to verify its feasibility. This work paves the way for providing a highly practicality sensor solution for the clinical application of MIPS theory.

## Materials & Methods

### Detection principle

**Figure 1:**
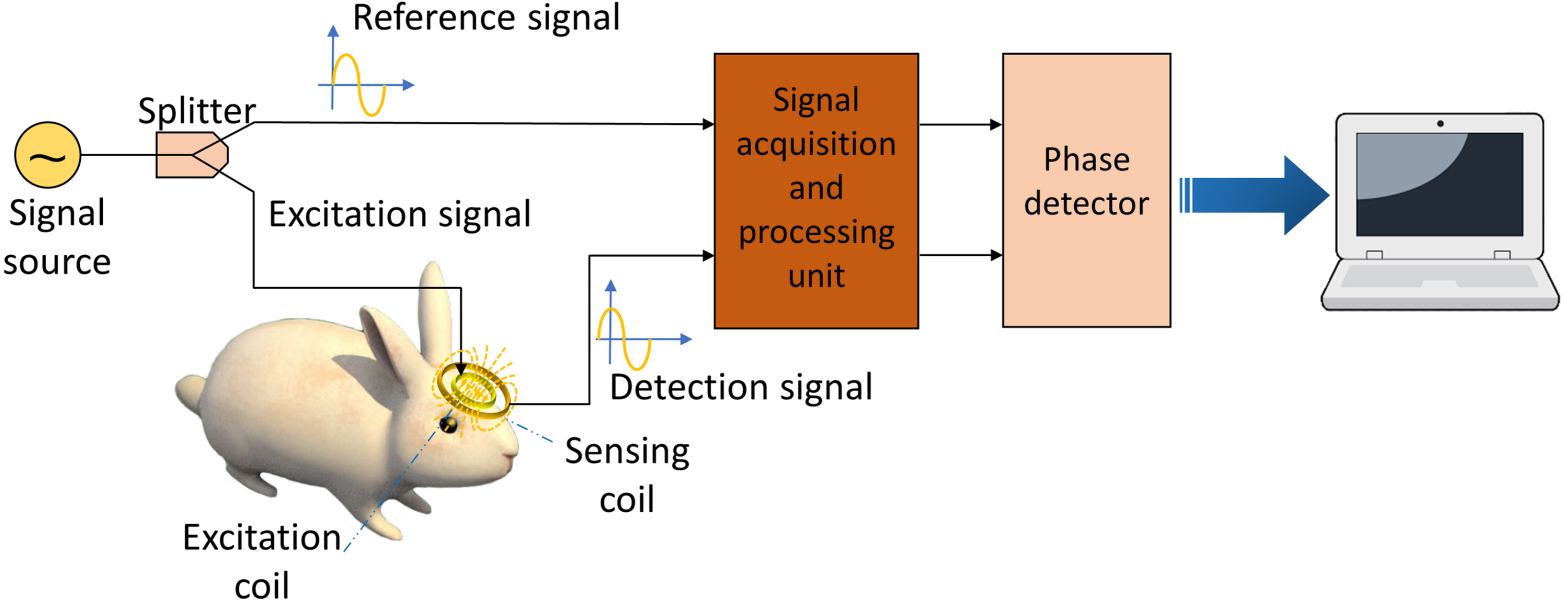
Schematic diagram of magnetic induction phase shift system.

Magnetic Induction Phase Shift (MIPS) monitoring is a non-invasive, real-time, continuous method to measure the conductivity change of an object. This method is based on the magnetic induction between the excitation-sensing coils and an object in its vicinity. Figure 1 sketched its basic physical principle. The signal source generates a sinusoidal signal and splits into two equivalent signals, one as reference signal and the other one as excitation signal sending to the excitation coil. The current-driven excitation coil sends out a primary alternating magnetic field (*B*) where this field penetrates the biological tissue in its vicinity and induces an eddy current. The secondary magnetic field (Δ*B*) generated by the eddy current is collected by the sensing coil, together with the primary magnetic field. There is a phase difference between the reference signal and detection signal. According to Griffiths, Stewart & Gough (1999), Δ*B* is related to *B* as follows:

(1) \begin{eqnarray*}\Delta B/B=Q\omega {\mu }_{0} \left[ \omega {\varepsilon }_{0} \left( {\varepsilon }_{r}-1 \right) -i\sigma \right] +R({\mu }_{r}-1)\end{eqnarray*}

where *ω* is the signal frequency, *σ*, ε_*r*_ and *μ*_*r*_ are the electrical conductivity, relative permittivity and relative permeability of the sample, separately. ε_0_ and *μ*_0_ are the permittivity and permeability of free space. *Q* and *R* are geometrical constants. Also, the magnetic field can be characterized by the voltage (*V*, Δ*V*) in the excitation and sensing coil (Griffiths, 2001; Griffiths, Stewart & Gough, 1999):

(2) \begin{eqnarray*}\Delta V/V=\Delta B/B.\end{eqnarray*}

Thus, the total signal (*V* + Δ*V*) collected by the sensing coil lags the primary signal by an angle *θ*. The change of phase angle *θ* is MIPS. The magnitude of ε_*r*_ in the MHz range is much lower than *σ*. Thus, there is a relationship between *θ* and the signal frequency *ω* and *σ* (Griffiths, 2001; Griffiths, Stewart & Gough, 1999):

(3) \begin{eqnarray*}\theta \propto \omega \sigma .\end{eqnarray*}

### Finite element method simulations

**Figure 2:**
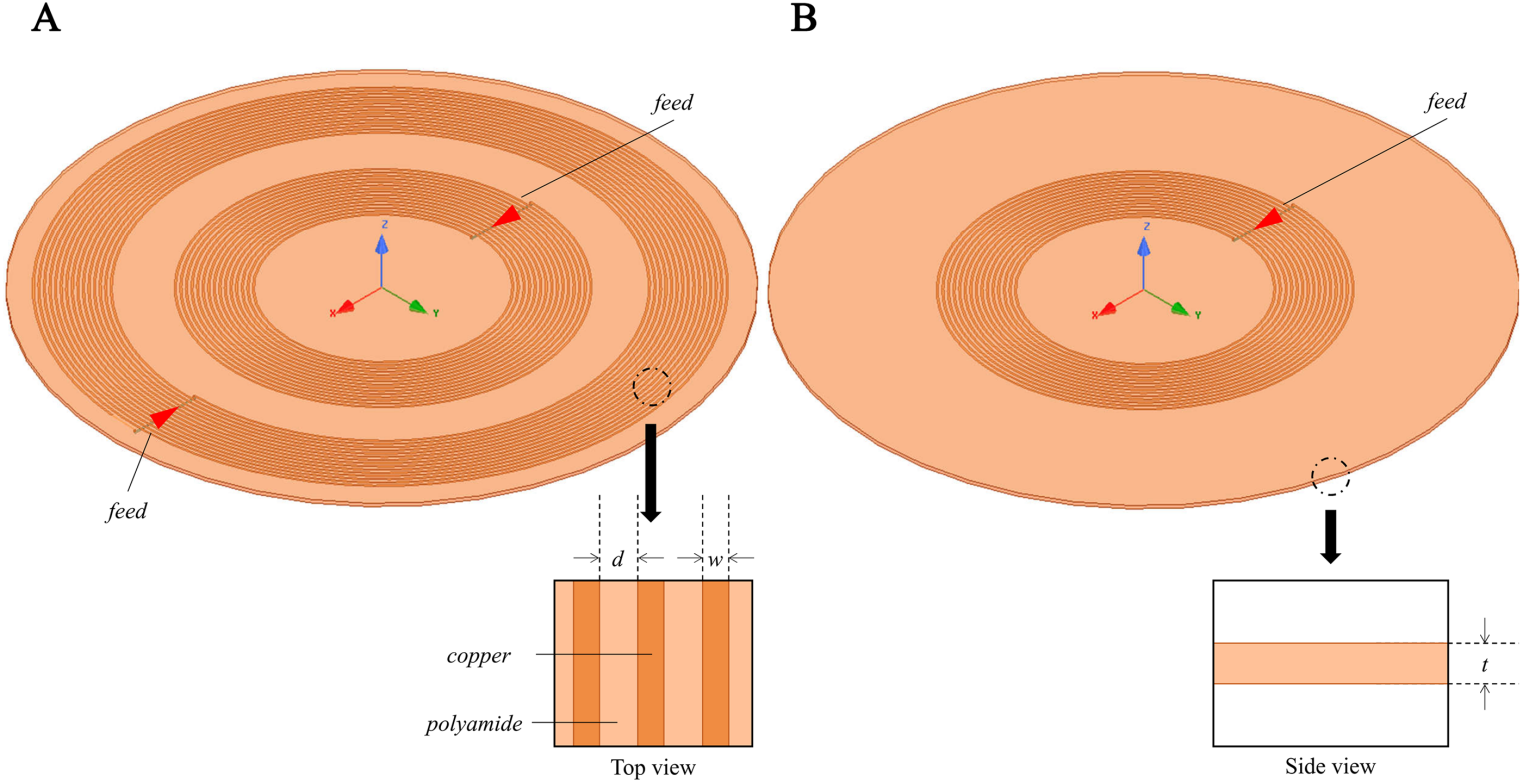
3D Simulation model for MIPS sensitivity map investigation. Conformal two-coil structure and single-coil structure.

**Figure 3:**
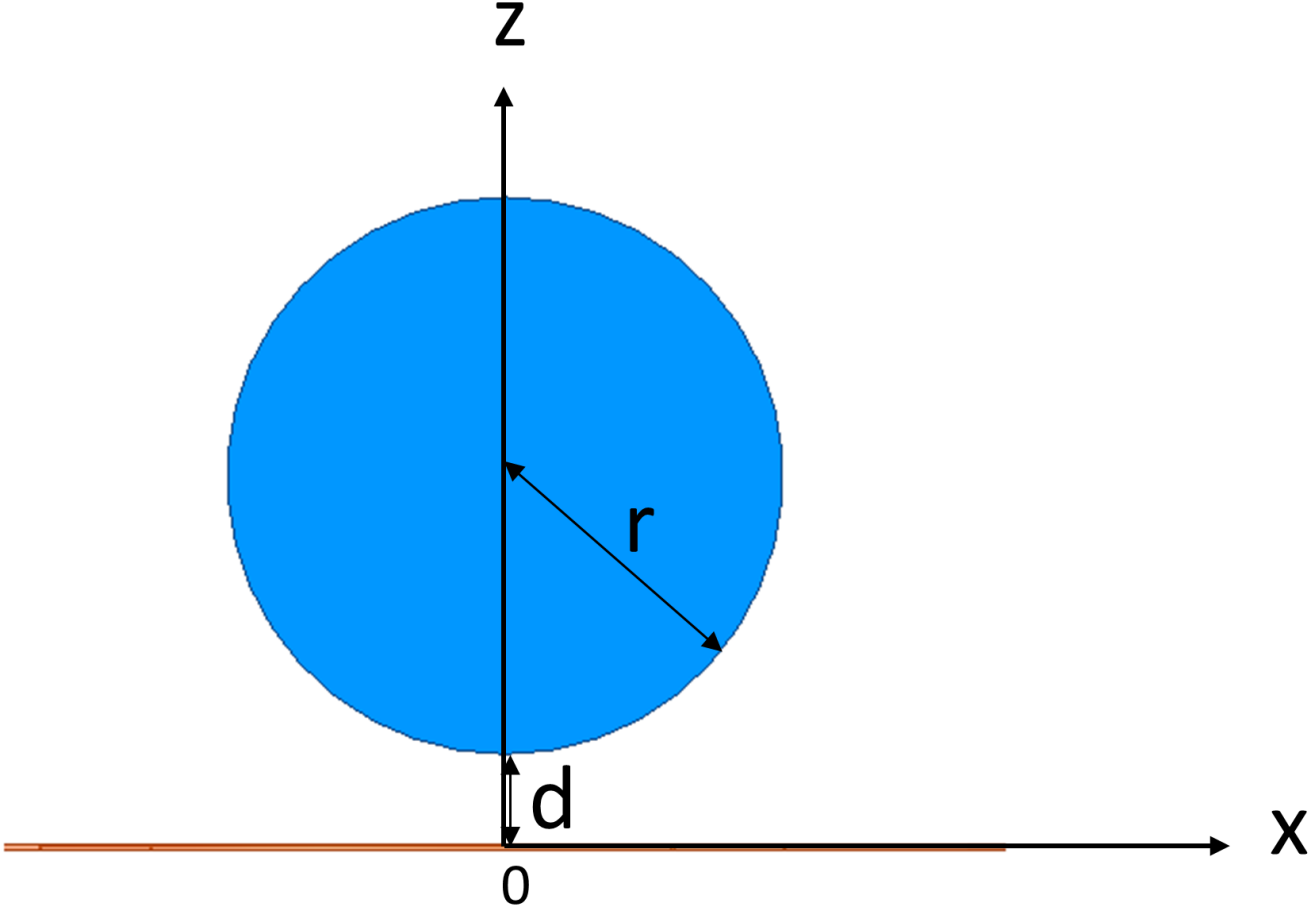
3D Simulation model for MIPS sensitivity map investigation. (A) Conformal two-coil structure. (B) Single-coil structure

In target to investigate the sensitivity map of the conformal two-coil and the single-coil structure, through which selecting a suitable coil designment for CE monitoring, simulation using the FEM was conducted using commercial software (HFSS, ANSYS Electronics Desktop 2018.0, USA). Two spiral coils were used to resemble a conformal two-coil structure, where the inner ring is excitation coil and the outer ring is sensing coil. Conformal refers specifically to the structure in which multiple coils are located on the same geometric plane, which comes from the planar compact Wireless Power Transfer (WPT) systems (Jonah, Geogakopoulos & Tentzeris, 2013; Hu & Georgakopoulos, 2017). The inner radius of excitation coil and sensing coil is 9 mm and 19 mm, separately (Fig. 2A). Each coil has 15 turns and with wire diameter (*w*) of 0.15 mm and line spacing (*d*) of 0.25 mm. Considering the skin effect of metal, the coil model in the simulation uses a plane model without thickness instead of a volume model. The boundary condition of coil was set as PEC. The sensor was developed on a 0.1-mm-thick (*t*) polyamide substrate, whose relative permittivity (ε_*r*_) and loss tangent are 3.2 and 0.003. Two coils were driven by lumped port excitation at 55 MHz. When cerebral edema occurs, the CSF will first be discharged from the cranial cavity due to compensation and followed by a certain range of brain blood compensation (Wykes & Vindlacheruvu, 2015). In the 1–100 MHz frequency band, the conductivity of intracranial cerebrospinal fluid (CSF) is the highest, followed by blood. Choosing the frequency of the system in this frequency band can better reflect the pathological process of CE. At the same time, the selection of this frequency is close to our previous work, in which we’ve conducted experiments using 65.5 MHz (Pan et al., 2015), 64.1 MHz (Zhao et al., 2019), 67.1 MHz (Yang et al., 2019a; Yang et al., 2019b). The two-coil sensor at the same lateral is a kind of traditional structure whereas it overcomes the disadvantages of using rigid materials to control the relative distance between excitation coil and sensing coil in practical applications. The conformal structure enables direct textile or FPC integration without affecting the relative position of coils. Subsequently, the outer coil was deleted and thus constructed a single-coil sensor (Fig. 2B), consistent with the structure used by Teichmann et al. (2015) and Teichmann et al. (2014). In both sensors, a 10 ml cylinder (radius *r* = 15 mm, height *h* ≈ 26.31 mm) was placed in the front center of the sensors. The initial distance between the center of beaker and center of substrates was *D* = *d* + *r* = 20 mm, as shown in Fig. 3. The boundary condition of the boundary of the analyzed domain was set as radiation. This cylinder was used to simulate the 1% saline solution with conductivity of 1.71 S/m.

Simulation investigation was carried out by displacing the 10 ml saline solution cylinder with a step of 1 mm from (0, 20) along the *x* axis and *z* axis. Through subtracting the phase of S21 at 55 MHz in free-space condition from those at all positions, MIPS sensitivity of the two-coil sensor at each position can be obtained. Likewise, through subtracting the phase of S11 at 55 MHz in free-space condition from those at all positions, MIPS sensitivity of the single-coil sensor can be obtained.

### Physical experiments

**Figure 4:**
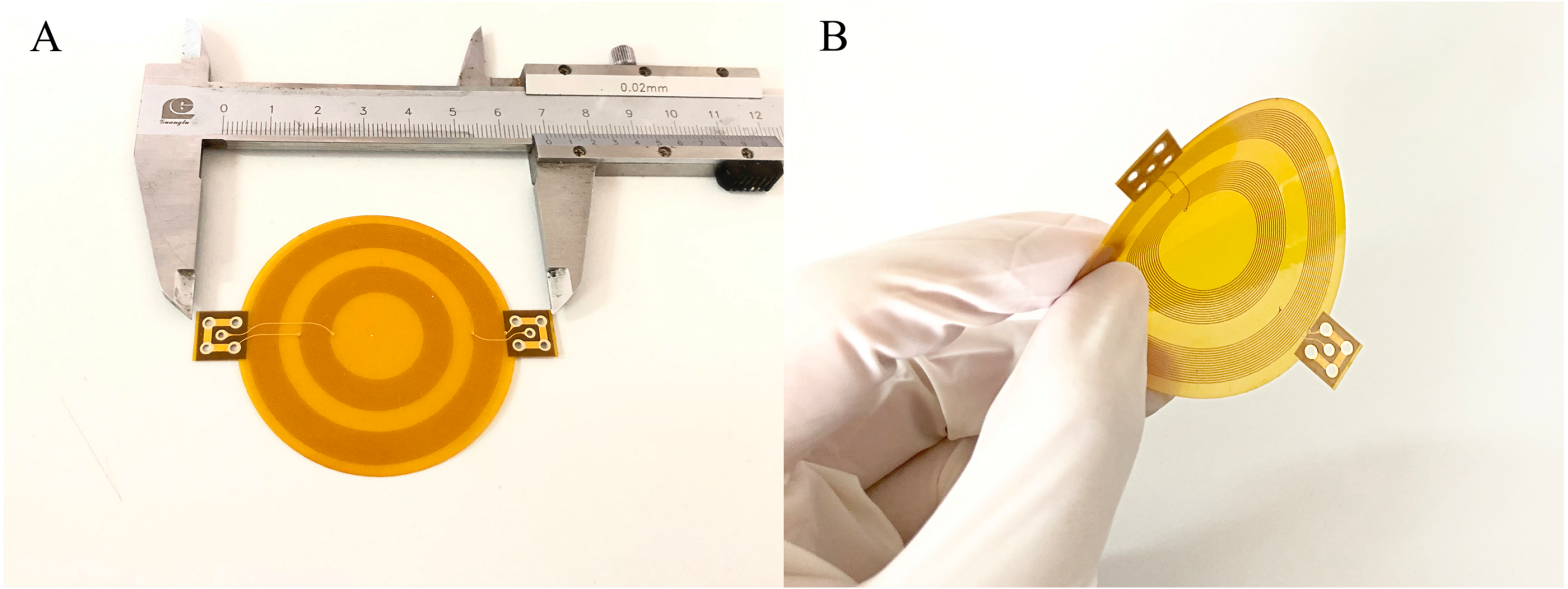
The flexible conformal MIPS sensor. (A) The geometric size is about 60 mm * 50 mm. (B) Bending deformation of sensors.

To investigate the effect of sensor bending on MIPS, flexible MIPS sensor was fabricated using FPC, as shown in Fig. 4. In this paper, a RF vector network analyzer (VNA, Agilent E5061B, USA) was utilized to realize MIPS detection. The output power of the VNA was 10dBm. The frequency band was set from 1 MHz to 100 MHz, with 1601 sample points and intermediate frequency bandwidth of 30 kHz. MIPS data were obtained from the phase of S21 at its characteristic frequency (53.7 MHz), where the transmission coefficient reach its maximum. The selection method of characteristic frequency comes from previous researches (Pan et al., 2015; Li et al., 2017a; Li et al., 2017b; Li et al., 2017c; Oziel et al., 2016). Physical and subsequent animal experiments were both carried out under those parameter settings. The characteristic frequency of animal experiments appeared at 57.7 MHz. In the three experiments, the system measurement frequency was slightly different. The detailed discussion on this phenomenon was arranged in the discussion part.

**Figure 5:**
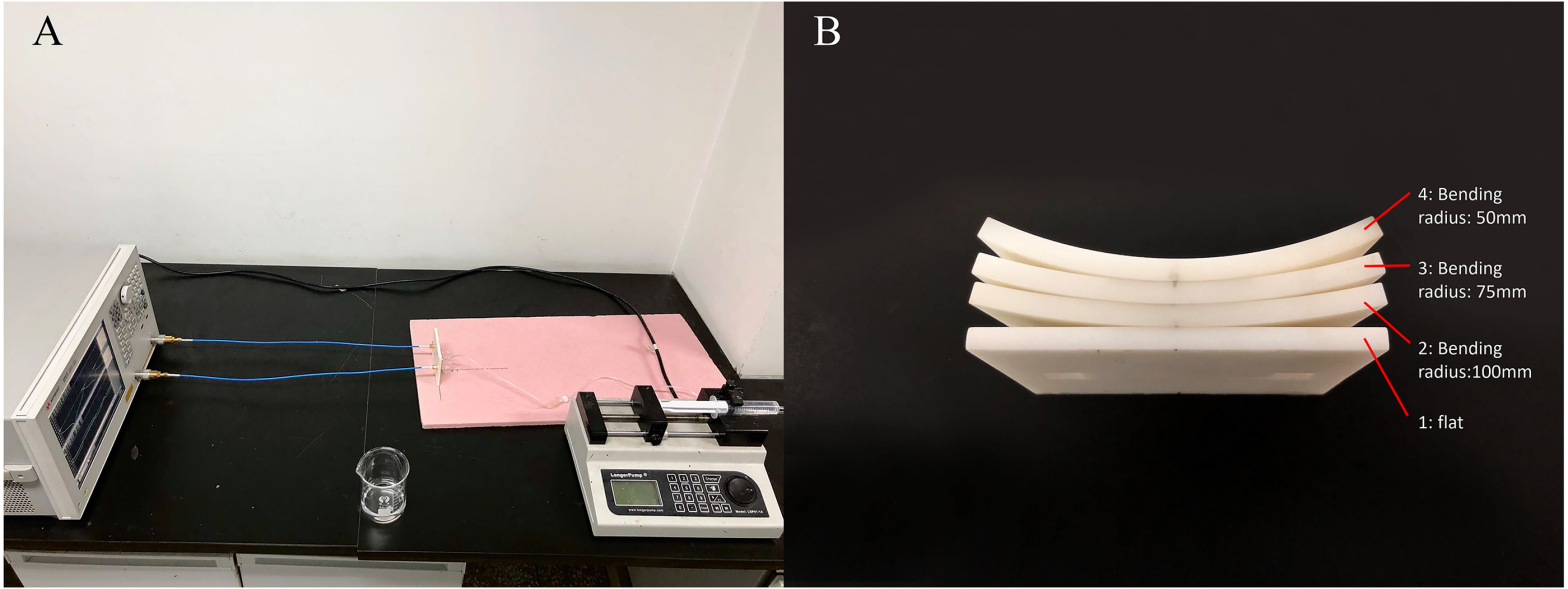
Experiments setup of physical experiments. (A) Physical experiment environment. (B) 3D-printed substrates with different bending radius.

The physical experiments were shown in Fig. 5. In this scenario, four 3D-printed substrates with different bending radius (flat, 100 mm, 75 mm, 50 mm) made of photosensitive resin were used for sensor fixing. Saline solution (1%) was used as the measured object. A beaker was fixed in front of the sensor to load the solution. Measurements utilizing each of those substrates was successively done while the distance between the outer ring of beaker and center point of substrates was kept at 5 mm (There was 4 mm between the mouth of the beaker and its inner wall), consistent with the settings of simulation experiments. Saline solution was injected into the beaker via injection pump (Longer, LSP01-1A, UK) from 0 ml to 10 ml with an increment of 1 ml, during which the MIPS data could be obtained. In each bending condition, the injection procedure was repeated for 20 times to get 20 sets of measurement data.

Subsequently, to verify the relationship between detection distance and sensor’s sensitivity, beaker was placed at another two positions for same measurements where the outer ring of the beaker is 10 mm and 15 mm from the center of the sensor, while the sensor was kept flat (using substrate 1).

### Animal experiments

In order to verify that the conformal MIPS sensor can monitor the development of CE, seventeen rabbits (available from Daping Hospital, 2.0–3.0 kg) were enrolled in the animal experiments. Rabbits were arrived one day before experiments and housed under ambient conditions (22 °C, 50% relative humidity, and a 12-h light/dark cycle), with free access to water and chow. These experiments were conducted under the guidance of the Administration of Animal Experiments for Medical Research Purpose issued by the Ministry of Health of China. The protocol was reviewed and approved by the Animal Experiments and Ethical Committee of Army Medical University (AMU, Chongqing, China), approval number AMUWEC2019237. All procedures were conducted while minimizing the suffering of rabbits. The rabbits were randomly divided into experimental group (*n* = 14) and control group (*n* = 3).

**Figure 6:**
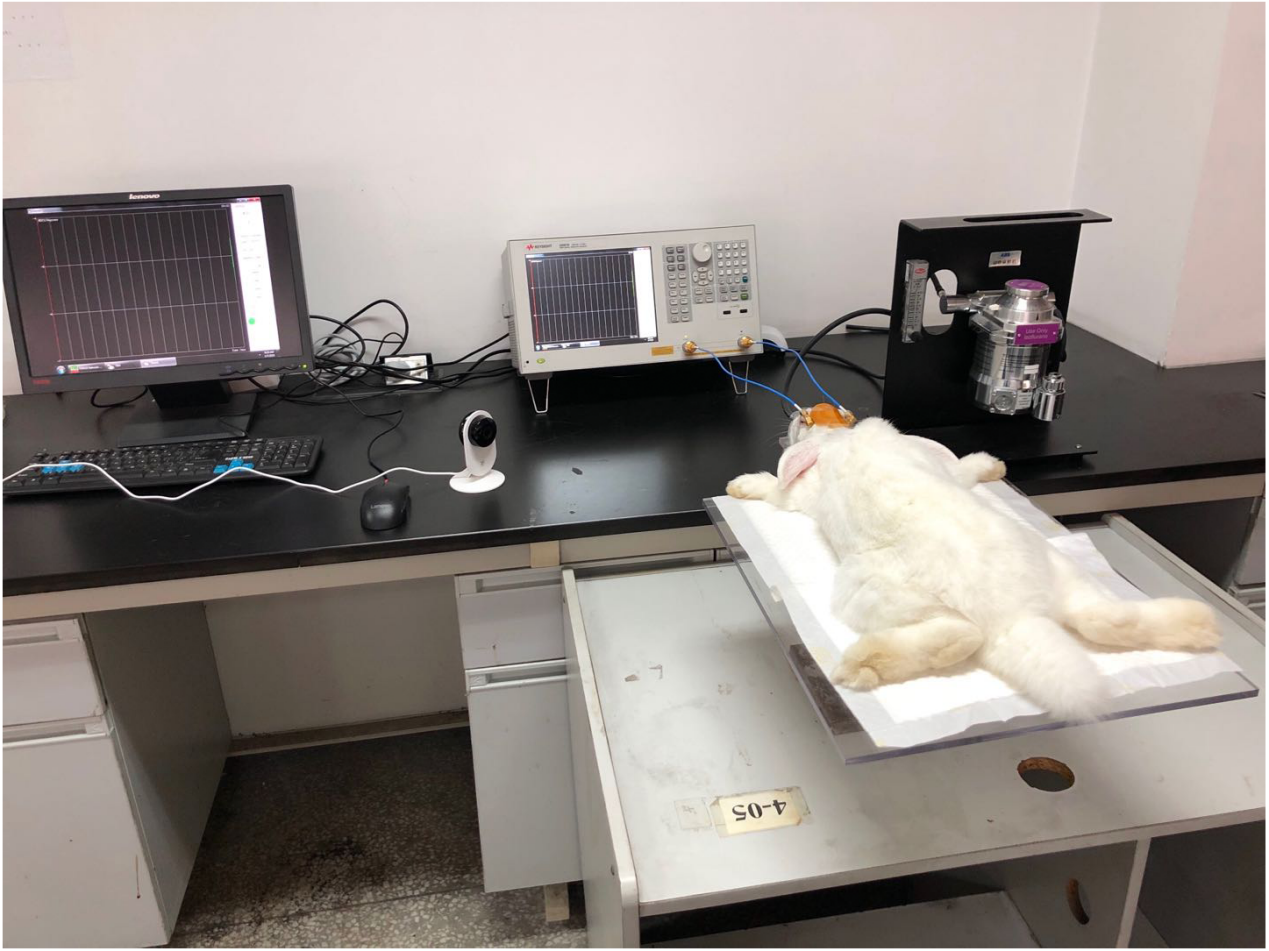
24 h real-time continuous monitoring experiments in rabbits.

Although it is of great practical significance to conduct research and data analysis on clinical CE patients, the complex pathological conditions and possible observation and treatment procedure shadows the data of clinical scenario. Thus, this experiment conduct animal models under controlled pathological conditions to better simulate the pathological progression of CE in a repeatable manner. Our previous researches had proved the validity of the CE model established by epidural freezing method (Kawai et al., 2003; Li et al., 2017a; Li et al., 2017b; Li et al., 2017c; Li et al., 2019). Rabbits were first anesthetized with pentobarbital (3%, 1 ml/kg) via ear vein. During surgery and monitoring, rabbits were given inhalation anesthesia (isoflurane, 1.5%) to maintain adequate anesthesia depth. After sedation, head and neck hair was removed. Then, rabbits underwent tracheal intubation for long-term monitoring purpose. After intubation, rabbits’ scalp was cut to expose the skull. Next, a bone window was drilled to expose the dura matter at 2 mm on the right side of sagittal suture and 2 mm on the posterior side of coronal suture. During drilling, the cryo-pencil was immersed into liquid nitrogen (−196 °C). After sufficient cold, the cryo-pencil were vertically put on the bone window for 120s. Then, the bone window was sealed using dental cement. In contrast, rabbits in control group experienced the same procedure but without freezing. After the establishment of CE and control model, rabbits were fit on the board and monitored for 24 h while the conformal MIPS sensor was placed close to the freezing point of the head. The characteristic frequency appeared at 57.7 MHz. The initial sampling rate was 12 times per hour. Rabbits in both groups were euthanized via IV pentobarbital overdose at the end of monitoring. Experimental arrangement was shown in Fig. 6.

### Signal processing and statistical analysis

Sensitivity results in simulation experiments was normalized to 0-1 for mapping and comparsion purpose. *S*_*t*_ Represented the normalized sensitivity of two-coil structure and *S*_*s*_ represented the normalized sensitivity of single-coil structure. The calculation formula of normalized sensitivity was given by:

(4) \begin{eqnarray*}& & {S}_{t} \left( x,y \right) = \frac{{S}_{t}^{\ast }(x,y)}{\max \nolimits \left( {S}_{t}^{\ast } \right) -\min \nolimits ({S}_{t}^{\ast })} \left\vert x=0,1,\ldots ,20,y=0,1,\ldots ,20 \right. \end{eqnarray*}

(5) \begin{eqnarray*}& & {S}_{s} \left( x,y \right) = \frac{{S}_{s}^{\ast }(x,y)}{\max \nolimits \left( {S}_{s}^{\ast } \right) -\min \nolimits ({S}_{s}^{\ast })} = \left\vert x=0,1,\ldots ,20,y=0,1,\ldots ,20 \right. \end{eqnarray*}

where ${S}_{t}^{\ast }$ and ${S}_{s}^{\ast }$ represents sensitivity values of two-coil structure and single-coil structure, respectively.

In order to quantitatively analyze the change trend of sensitivity, *R*_*t*_ and *R*_*s*_ were used to represent the ratio of the coordinate points of the normalized sensitivity in a specific interval to all the measured coordinate points. The value of *R*_*t*_ and *R*_*s*_ were given by:

(6) \begin{eqnarray*}& & {R}_{t}= \frac{n}{21\ast 21} \ast 100\text{%}, i=1,2,\ldots ,n \left\vert {S}_{t} \left( x,y \right) \geq a \right. \end{eqnarray*}

(7) \begin{eqnarray*}& & {R}_{s}= \frac{n}{21\ast 21} \ast 100\text{%}, i=1,2,\ldots ,n \left\vert {S}_{s} \left( x,y \right) \geq a \right. \end{eqnarray*}

where *n* represents the total number higher than the threshold a.

In animal experiments, the initial sampling rate of the MIPS signal was 12 times/h. Considering that the original signal was mixed with heart and lung activities plus external interference, a wavelet transform was used to reduce noise. Data processing was performed by MATLAB R2015a (MathWorks, Inc., USA). Moreover, The physical and animal experimental data were statistically analyzed utilizing nonparametric multi-independent/independent sample test and two-sample independent *t*-test, seperately. Statistical analyses were performed by SPSS 19.0 (SPSS Inc., Chicago, IL, USA). The significance level was set at *p* < 0.05.

## Results

### Results of simulation experiments

**Figure 7:**
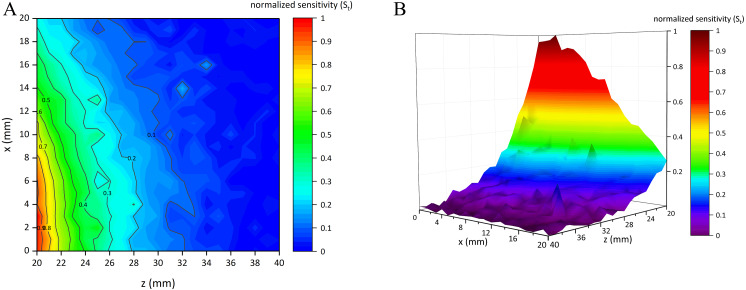
Normalized sensitivity map of conformal two-coil sensor structure. (A) 2D-view of the sensitivity distribution. (B) 3D-view of the sensitivity distribution.

**Figure 8:**
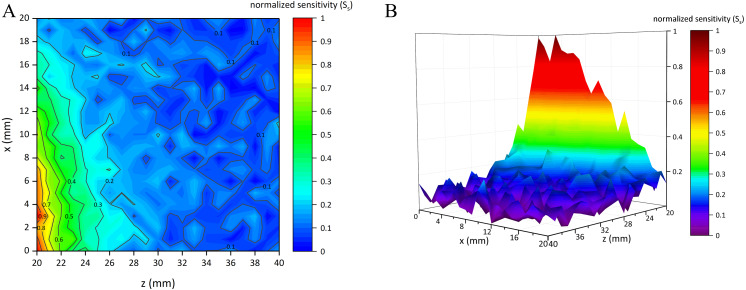
Normalized sensitivity map of single-coil sensor structure. (A) 2D-view of the sensitivity distribution. (B) 3D-view of the sensitivity distribution.

Figure 7 shows the normalized sensitivity map of the conformal two-coil sensor structure (*S*_*t*_ ∈ [0, 1]). It can be found that this structure had the characteristic of local focus, where the sensitivity got higher when close to the center. The highest sensitivity (*S*_*t*_ = 1) appeared at (*x*, *z*) = (3, 20). *S*_*t*_ gradually attenuated form the center outward. Figure 8 shows the normalized sensitivity map of the single-coil sensor structure (*S*_*s*_ ∈ [0, 1]). The highest sensitivity (*S*_*s*_ = 1) also appeared at (*x*, *z*) = (3, 20). Both sensitivity map of the two sensor structures showed a manner of elliptical shape, where their sensitivity attenuated sharper axially. Especially, *S*_*s*_ attenuated faster both axially and radially than that of two-coil structure. Moreover, as shown in Fig. 7, *S*_*t*_ is better distributed while there are many burrs in Fig. 8. Table 1 lists the sensitivity attenuation trend in both two sensor structures, which further confirmed the fact that the *S*_*s*_ was more concentrated near the center and attenuated quickly. In comparison, *R*_*t*_ is gentler, with more proportion up to ≥0.3 level. Due to the burrs in the low-sensitivity area of Fig. 8, *R*_*s*_ in ≥0.1 interval was higher. It can be concluded that single-coil structure also has the characteristic of local focus whereas its flatness and uniformity of sensitivity map is poorer. Based on this conclusion, MIPS sensor with the two-coil structure was selected and fabricated in physical experiments.

**Table 1:** Proportion of normalized sensitivity at each interval.

Sensitivity range	*R*_*s*_	*R*_*t*_
≥0.9	0.91%	1.59%
≥0.7	3.18%	4.54%
≥0.5	7.03%	9.75%
≥0.3	16.10%	21.32%
≥0.1	68.03%	50.79%

### Results of physical experiments

**Figure 9:**
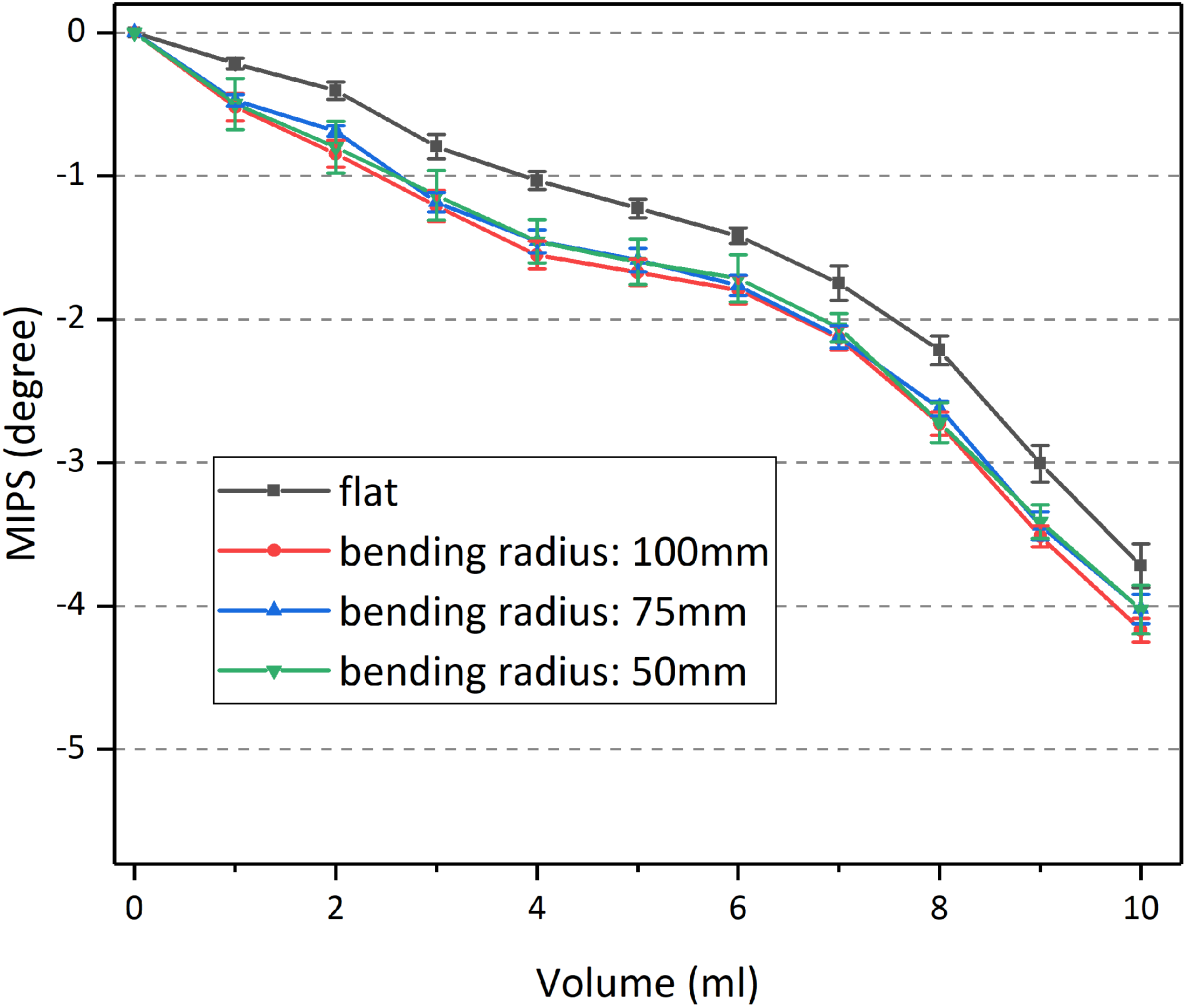
Results of MIPS vs injection volume under different bending radius.

Figure 9 demonstrates the MIPS at 53.7 MHz as a function of injection volume (mean ± SD), in which four color lines indicated four bending radius conditions, respectively. Obviously, the MIPS data showed a downward trend with increasing volume, indicating that MIPS can reflect the conductivity change of the measured object.

To quantitatively study whether bending is related to the sensor’s performance, the MIPS data under three volumes (3 ml, 6 ml, 9 ml) were extracted for statistical analysis. First, those data were enrolled for nonparametric multi-independent sample test (Kruskal Wallis test), as shown in Table S1. It can be found that MIPS under different bending radius in all of the three volumes were statistically significant (*p* < 0.05). This shows that at any volume, there was a significant difference in those data. But the relationship between the data within each group remains unclear. Thus, Non-parametric independent sample test was conducted between each bending radius (Table S2).

**Figure 10:**
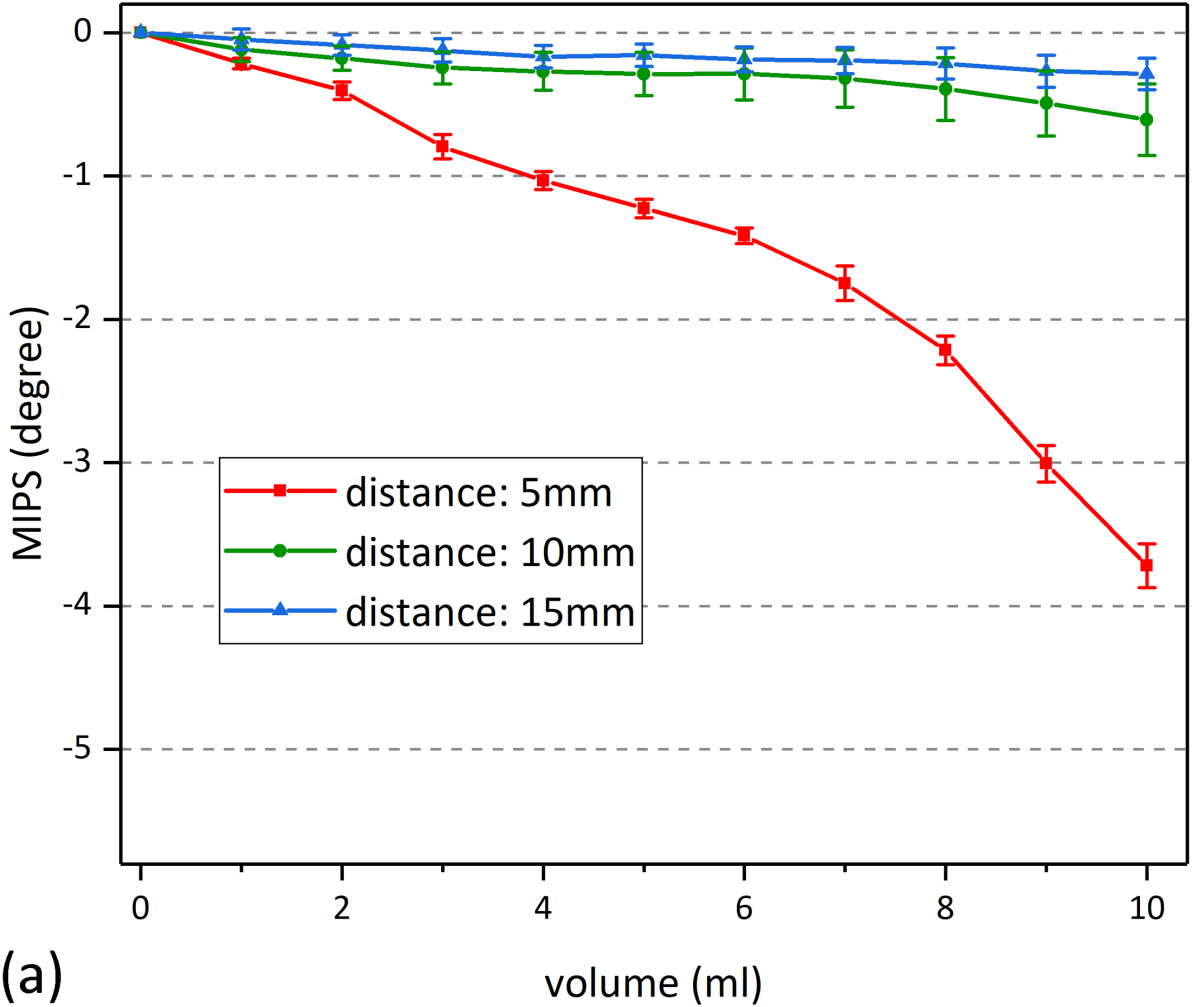
MIPS data under different detection distance. (A) MIPS trend as a function of volume. (B) Maximum change of MIPS in each detection distance.

Statistical data shows that, in all of the three volume, there was no significant difference between different bending radius (2-3/2-4/3-4) (*p* > 0.05), which means that the performance of the sensor is independent of its bending degree. Besides, MIPS in each of the three bending cases was significantly different from the flat one (1-2/1-3/1-4) (*p* < 0.05). That can also be found in Fig. 9 that the MIPS data under those bending conditions was slightly larger. This may be caused by the reduced gap between the sensor and beaker, as a result of bending. Smaller gaps were provided with higher *S*_*t*_. Figure 10 shows the MIPS changes under three detection distance (using substrate 1). As shown below, the sensitivity of the MIPS did decay with increasing distance, consistent with the simulation results where *S*_*t*_ attenuated quickly along its axial direction.

### Results of animal experiments

**Figure 11:**
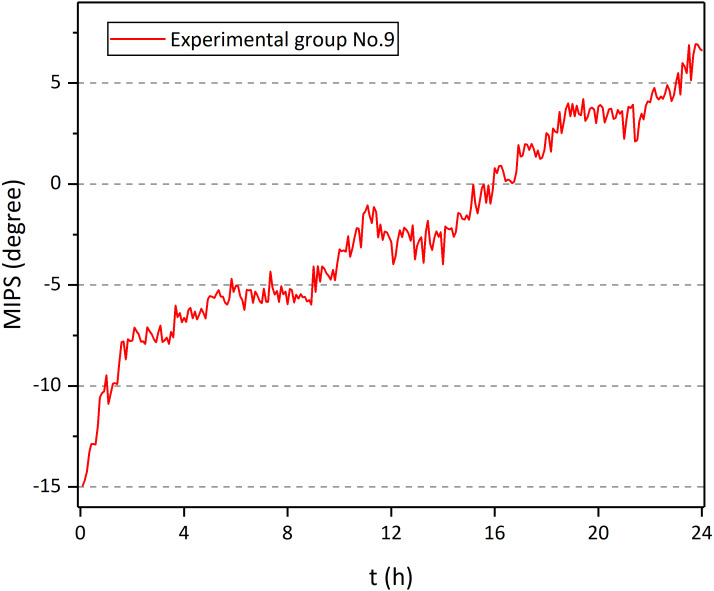
MIPS of No. 9 rabbit in experiment group as a function of time.

**Figure 12:**
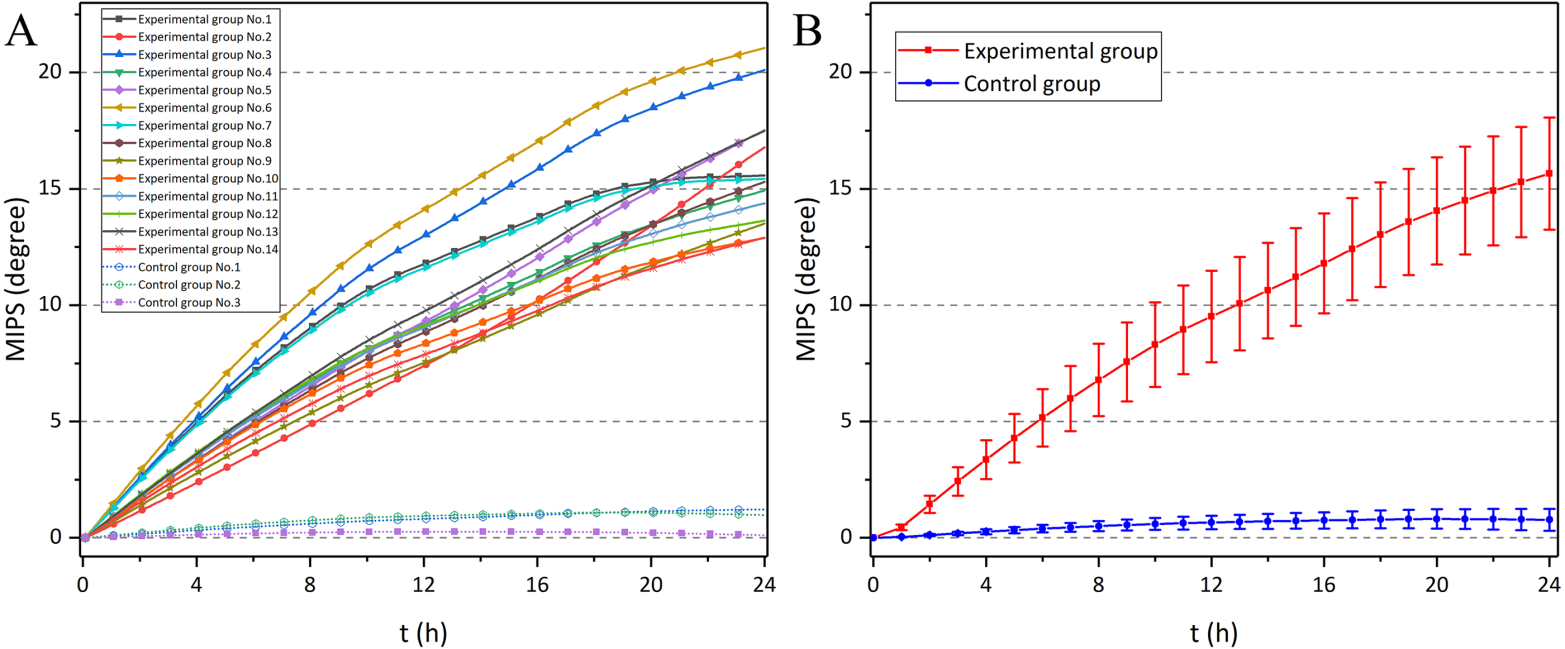
The MIPS of 24 h monitoring experiments in experimental group and control group, as a function of time. (A) MIPS in each rabbits. (B) Mean ± SD MIPS in each group (error bars with hourly data).

Figure 11 shows the MIPS data at 57.7 MHz of the 9th rabbit in the experimental group. There was a clear upward trend over time. Also, it can be found that the raw data was doped with breathing, head movement and external interference, which influenced the actual trend of MIPS-characterized CE. Therefore, the original data is processed through wavelet transform. Figure 12 shows the MIPS change at 57.7 MHz in 24 h monitoring of rabbits in experimental group and control group. For mapping needs, the initial MIPS value of all data was normalized to 0. Rabbits in experimental group all showed a significant upward trend with maximum change at 24th hour (15.66 ± 2.41°). After the same surgical procedures, the development of CE seemed to have individual differences. In contrast, rabbits in control group merely had a slight increase in MIPS and kept stable with insignificant variation in the whole 24 h measurement (0.78 ± 0.47° at 24th hour). Our previous finding showed a 24 h downward trend of the MIPS with largest change of −13.1121 ± 2.3953° at the 24th hour in CE group and no obvious trend in the control group (−0.87795 ± 1.5146° at 24th hour). This conformal MIPS sensor is consistent with previous studies in monitoring and distinguishing CE (Li et al., 2017a; Li et al., 2017b; Li et al., 2017c; Zhao et al., 2019; Li et al., 2019), whereas the change trend of MIPS in the experimental group was opposite.

Then, the differences between the experimental group and the control group were statistically analyzed (Table 2). Through two-sample independent *t*-test, it can be found that those two groups showed significant difference from the 1st hour. The early diagnostic ability of the conformal MIPS sensor in this study was the same as that in previous study by Li et al. (2017a), Li et al. (2017b) and Li et al. (2017c).

**Table 2:** Two-sample independent *t*-test of experimental group & control group.

Time	Sample	Variances	*F*	Significance	*T*	Significance (Two-tailed)	95% Confidence interval of the difference	
							Lower	Upper
1 h	Experiment group vs Control group	Equal variances assumed	4.39	0.05	5.83	3.29e^−5^	0.26	0.57
			Equal variances not assumed			12.34	3.50e^−9^	0.34	0.49
6 h			Equal variances assumed	4.29	0.05	6.26	1.52e^−5^	3.14	6.39
			Equal variances not assumed			13.19	1.32e^−9^	4.00	5.54
12 h			Equal variances assumed	3.78	0.07	7.32	2.52e^−6^	6.28	11.43
			Equal variances not assumed			15.23	1.59e^−10^	7.62	10.09
18 h			Equal variances assumed	3.15	0.09	8.81	2.57e^−7^	9.28	15.19
			Equal variances not assumed			17.95	1.67e^−11^	10.78	13.69
24 h			Equal variances assumed	2.75	0.12	10.00	4.97e^−8^	11.71	18.05
		Equal variances not assumed			19.93	6.38e^−12^	13.29	16.48

## Discussion

As stroke is governed by many factors, such as blood gas concentrations, blood–brain barrier (BBB), reperfusion, underlying vascular anatomy (Howells et al., 2010), investigating the whole brain’s conductivity change may also introduce too much irrelevant physiological information which affects the pertinence between the MIPS data with pathophysiological changes of the lesions. MIPS sensor or multi-sensor array has seldomly tackled the problem that to achieve local focus measurement. Based on this, we designed a flexible conformal MIPS sensor that is better suited for targeting local change of dielectric properties which reflects the CE progression. More importantly, Selim & Norton (2020) have suggested that investigation and interventions targeting CE should be conducted in the early stage (within hours of ICH onset) and maintained for several days. A user-friendly solution between monitoring modality and patients is critical for long-term practice. Nevertheless, most MIPS sensors previously reported were made of hard materials, which is not suitable to monitor CE in long time, especially for critically ill patients. For these, the flexible MIPS sensor was manufactured by FPC materials. This sensor is not only consistent with the requirements of clinical CE monitoring, but also can be more trouble-free for medical staff without relevant expertise in engineering.

In simulation and physical experiments, both single-coil structure and two-coil structure have the characteristic of local focus. But with the increase of axial distance or radial distance, *S*_*s*_ decays faster than *S*_*t*_. That infers that the two-coil structure can achieve deeper measurement depth under the same geometric parameter design, which provides an optimized choice for subsequent sensor design targeting different brain regions considering the measurement depth of single-coil structure is hindered by its characteristic. In addition, to accurately obtain CE information, it’s a better choice to explore several specific brain regions. The MIPS sensors with local focus characteristics provides more specific information if sensor or multi-sensor array was placed at proper measure position. Especially, in clinical application scenarios, cerebral stroke has several subtypes and there are complex transformation mechanisms between those various subtypes. A specific coil design or coil array study may have high applicability to certain disease subtypes and even some other detections of vital signs or diseases. Xiao, Tan & Dong (2019) proposed an open cambered MIT system, in which they rearranged the coil array and optimized the near-subsurface sensing, specifically improved the sensitivity of extra axial hemorrhage. In order to investigate the impedance change of cervical tissue during pregnancy, Wang et al. (2017) developed a ferrite-cored coaxial gradiometer magnetic probe which eliminate the interfere signals from surrounding tissue and eventually confined the sensitivity to the volume defined by the gap between the ferrite core and outer tube. Furthermore, the flexible conformal MIPS sensor enables clinical readiness. Flexible sensors benefit from their material properties and are therefore suitable as clinical sensor carriers. The FPC-based conformal sensor can be “attached” to the patient’s head without being fixed by rigid materials like the conventional coaxial coil which is susceptible to head movement. On the premise of imaging data acquisition, the relative position of sensors can be adjusted flexibly through which accurately extracting the information of CE. Plus, it’s necessary to investigate the influence of the sensor’s geometric parameters and shape on the sensitivity map, through which we can select the appropriate parameters to design a sensor that meets clinical requirements.

As with the development of CE, the intracranial components, volume, and cellular metabolism level happens to change, which, in turns, gradually result in the increase of the conductivity of the brain tissue (Fujita, Ueda & Yagi, 1972). Theoretically, MIPS is negatively correlated with conductivity, as shown in those results of physical experiments. However, The MIPS data obtained by this sensor showed an upward trend, which was positively correlated with the severity of cerebral edema in rabbits. That apparently was opposite with that of physical experiment. This, on the one hand, may be caused by different measurement frequencies. In 2003, Goss et al. discussed that there were various inductive and capacitive coupling mechanisms in electromagnetic tomography (Goss et al., 2003). Different measured objects had different coupling parameters in this two-port network structure. In physical experiments, the characteristic frequency appeared at 53.7 MHz, at which the signal transmission power was the largest. In animal experiments, the characteristic frequency appeared at 57.7 MHz. Flores, Rubinsky & Gonzalez (2008) had reported that there were frequencies where the phase shift may turn to opposite way. On the other hand, Whether the measured object is a single or a multilayer medium also has an effect on the trend of the result. In 2004, Hollaus et al. found that the imaginary part of the sensitivity showed opposite trend when the agar sphere was in free space and immersed in the saline (Hollaus et al., 2004). In point of fact, magnetic induction models for multilayer medium may require more complex derivation and verification. In physical experiments, a measurement model with a single controllable independent variable was used to test and verify the sensor. Effective measurement data showed the correlation between MIPS measurement method and the change of dielectric parameters. But it is true that the actual brain edema model is controlled by multiple parameters. Analyzing the data characteristics of a specific cellular damage type in the brain may also require a large amount of measurement data, and the use of data algorithms such as machine learning.

The CE model we built based on epidural freezing method mainly triggered vasogenic CE accompanied by cytotoxic CE. Current non-invasive monitoring method (MIPS, VIPS, EIT etc.) mainly focus on the differentiation of cerebral hemorrhage/cerebral ischemia, stroke diagnosis and monitoring, etc. Few studies focused on identifying CE subtypes. Different CE types have different treatment options (Michinaga & Koyama, 2015). Vasogenic edema is defined as extracellular accumulation of fluid resulting from disruption of the BBB and extravasations of serum proteins, while cytotoxic edema mainly expresses as cell swelling caused by intracellular accumulation of fluid. Because BBB disruption is a reversible process, it is possible to recover under medical treatment (VEGFs, MMP9, and other inhibitors). On the other hand, anti-cytotoxic edema drugs are also expected to improve the abnormal outflow of intravascular fluid. Obviously, it would be valuable for the distinction between vasogenic & cytotoxic CE. Due to the limitation of experimental scale and CE model, this study has not conducted in-depth study on this issue, but the distinction between different CE subtypes based on MIPS data is a promising research direction.

Early identification of cerebral edema is a tough problem amid there is no fully accepted method for bedside, early-stage diagnosis method. Proper clinical intervention in early stage of CE helps improve prognosis and prevent complications. Our previous researches illustrated that the MIPS method can achieve early identification of ICP rising and the pathophysiological changes of the brain (Sun et al., 2019; Li et al., 2019; Zhao et al., 2019). Subsequent studies will focus on establishing the predicting model for CE based on advanced data processing and machine learning.

## Conclusions

MIPS technology has advantages of noninvasiveness, noncontact, good portability, and real-time continuous bedside monitoring. Based on its theory, this study proposed a flexible conformal MIPS sensor for collecting accurate information of reginal CE. In the simulation experiment, conformal two-coil structure is more suitable for detection of regional CE compared with single-coil structure on account of its better flatness and uniformity of sensitivity map. Physical experiment results showed that the flexible MIPS sensor fabricated based on this structure has a high focusing performance. In addition, its ability of being robust to bending makes it possible to freely adjust placement to the optimal detection area for the intracranial lesion without moving the patient’s head, which contributes to improving the accuracy and consistency of clinical CE monitoring. The 24-hour data of MIPS in rabbits indicated that the flexible conformal MIPS sensor can monitor local CE effectively in long-time. However, this is only a preliminary study. We will devote to the design of flexible multi-coil sensor array for extracting more precise and plentiful information in multiple specific brain regions. Furthermore, it is expected to establish a predicting model of CE using advanced data processing and machine learning methods on the basis of the follow-up studies.

##  Supplemental Information

10.7717/peerj.10079/supp-1 Table S1 Nonparametric multi-independent sample test of MIPS data as a function of bending radius at three volume

10.7717/peerj.10079/supp-2 Table S2 Non-parametric independent sample test of MIPS data between any pare of bending radius in each volume Group 1/2/3/4 respectively represent flat and bending radius of 100 mm, 75 mm, 50 mm

10.7717/peerj.10079/supp-3 Data S1 Raw data of physical experiments, simulation experiments and animal experiments

## Additional Information and Declarations

### Data Availability

The following information was supplied regarding data availability: The raw measurements are available as a Supplemental File.

## References

[ref-1] Chen M, Ma Y, Li Y, Wu D, Zhang Y, Youn CH (2017). Wearable 2.0: enabling human-cloud integration in next generation healthcare systems. IEEE Communications Magazine.

[ref-2] Cook AM, Morgan Jones G, Hawryluk GW, Mailloux P, McLaughlin D, Papangelou A, Samuel S, Tokumaru S, Venkatasubramanian C, Zacko C (2020). Guidelines for the acute treatment of cerebral edema in neurocritical care patients. Neurocritical Care.

[ref-3] Dhar R, Chen Y, An H, Lee J (2018). Application of machine learning to automated analysis of cerebral edema in large cohorts of ischemic stroke patients. Frontiers in Neurology.

[ref-4] Flores O, Rubinsky B, Gonzalez CA (2008). Experimental sensitivity study of inductive phase shift spectroscopy as non-invasive method for hypoperfusion vs bleeding volumetric detection in brain.

[ref-5] Fujita S, Ueda T, Yagi M (1972). Detection of experimental and clinical brain edema using an electrical impedance method. Journal of Neurosurgery.

[ref-6] González CA, Horowitz L, Rubinsky B (2007). In vivo inductive phase shift measurements to detect intraperitoneal fluid. IEEE Transactions on Biomedical Engineering.

[ref-7] González CA, Pérez M, Hevia N, Arámbula F, Flores O, Aguilar E, Hinojosa I, Joskowicz L, Rubinsky B (2010). Over-hydration detection in brain by magnetic induction spectroscopy.

[ref-8] González CA, Rubinsky B (2006). A theoretical study on magnetic induction frequency dependence of phase shift in oedema and haematoma. Physiological Measurement.

[ref-9] Gonzalez CA, Valencia JA, Mora A, Gonzalez F, Velasco B, Porras MA, Salgado J, Polo SM, Hevia-Montiel N, Cordero S (2013). Volumetric electromagnetic phase-shift spectroscopy of brain edema and hematoma. PLOS ONE.

[ref-10] Goss D, Mackin RO, Crescenzo E, Tapp HS, Peyton AJ (2003). Understanding the coupling mechanism in high frequency EMT.

[ref-11] Griffiths H (2001). Magnetic induction tomography. Measurement Science and Technology.

[ref-12] Griffiths H, Stewart WR, Gough W (1999). Magnetic induction tomography: a measuring system for biological tissues. Annals of the New York Academy of Sciences.

[ref-13] He W, Luo H, Xu Z, Wang J (2010). Multi-channel magnetic induction tomography measurement system.

[ref-14] Hollaus K, Magele C, Merwa R, Scharfetter H (2004). Numerical simulation of the eddy current problem in magnetic induction tomography for biomedical applications by edge elements. IEEE Transactions on Magnetics.

[ref-15] Hopfer M, Planas R, Hamidipour A, Henriksson T, Semenov S (2017). Electromagnetic tomography for detection, differentiation, and monitoring of brain stroke: a virtual data and human head phantom study. IEEE Antennas & Propagation Magazine.

[ref-16] Howells DW, Porritt MJ, Rewell SS, O’Collins V, Sena ES, Van Der Worp HB, Traystman RJ, Macleod MR (2010). Different strokes for different folks: the rich diversity of animal models of focal cerebral ischemia. Journal of Cerebral Blood Flow & Metabolism.

[ref-17] Hu H, Georgakopoulos SV (2017). Multiband and broadband wireless power transfer systems using the conformal strongly coupled magnetic resonance method. IEEE Transactions on Industrial Electronics.

[ref-18] Jha RM, Kochanek PM, Simard JM (2019). Pathophysiology and treatment of cerebral edema in traumatic brain injury. Neuropharmacology.

[ref-19] Jin G, Sun J, Qin M, Tang Q, Xu L, Ning X, Xu J, Pu X, Chen M (2014). A new method for detecting cerebral hemorrhage in rabbits by magnetic inductive phase shift. Biosensors and Bioelectronics.

[ref-20] Jonah O, Geogakopoulos SV, Tentzeris MM (2013). Strongly coupled wireless power transfer with conformal structures.

[ref-21] Kalkonde YV, Alladi S, Kaul S, Hachinski V (2018). Stroke prevention strategies in the developing world. Stroke.

[ref-22] Kawai N, Kawanishi M, Okada M, Matsumoto Y, Nagao S (2003). Treatment of cold injury-induced brain edema with a nonspecific matrix metalloproteinase inhibitor MMI270 in rats. Journal of Neurotrauma.

[ref-23] Kellner CP, Sauvageau E, Snyder KV, Fargen KM, Arthur AS, Turner RD, Alexandrov AV (2018). The VITAL study and overall pooled analysis with the VIPS non-invasive stroke detection device. Journal of NeuroInterventional Surgery.

[ref-24] Koo HR, Lee YJ, Gi S, Khang S, Lee JH, Lee JH, Lim MG, Park HJ, Lee JW (2014). The effect of textile-based inductive coil sensor positions for heart rate monitoring. Journal of Medical Systems.

[ref-25] Kumar BS, Athar MD, Kamran M (2017). Management of severe TBI-a review of recent literature. JHN Journal.

[ref-26] Li G, Chen J, Gu S, Yang J, Chen Y, Zhao S, Xu J, Bai Z, Ren J, Xu L (2019). A dual parameter synchronous monitoring system of brain edema based on the reflection and transmission characteristics of two-port test network. IEEE Access.

[ref-27] Li G, Ma K, Sun J, Jin G, Qin M, Feng H (2017a). Twenty-four-hour real-time continuous monitoring of cerebral edema in rabbits based on a noninvasive and noncontact system of magnetic induction. Sensors.

[ref-28] Li G, Sun J, Ma K, Yan Q, Zheng X, Qin M, Jin G, Ning X, Zhuang W, Feng H, Huang S (2017b). Construction of a cerebral hemorrhage test system operated in real-time. Scientific Reports.

[ref-29] Li G, Zheng X, Sun J, Ma K, Jin G, Feng H, Qin M (2017c). A non-invasive non-contact continuous monitoring system of brain edema based on magnetic induction phase shift and computer programming. Nanoscience and Nanotechnology Letters.

[ref-30] Luo HJ, He W, Xu Z, Liu L (2012). Preliminary results on brain monitoring of meningitis using 16 channels magnetic induction tomography measurement system. Progress in Electromagnetics Research.

[ref-31] Michinaga S, Koyama Y (2015). Pathogenesis of brain edema and investigation into anti-edema drugs. International Journal of Molecular Sciences.

[ref-32] Murthy SB, Moradiya Y, Dawson J, Lees KR, Hanley DF, Ziai WC, Butcher K, Davis S, Gregson B, Lyden P, Mayer S, Muir K, Steiner T (2015). Perihematomal edema and functional outcomes in intracerebral hemorrhage. Stroke.

[ref-33] Olindo S, Sibon I, Badaut J, Plesnila N (2017). Chapter 22—cerebral edema in cerebrovascular diseases. Brain edema.

[ref-34] Oziel M, Hjouj M, Gonzalez CA, Lavee J, Rubinsky B (2016). Non-ionizing radiofrequency electromagnetic waves traversing the head can be used to detect cerebrovascular autoregulation responses. Scientific Reports.

[ref-35] Oziel M, Hjouj M, Rubinsky B, Korenstein R (2020). Multifrequency Analysis of single inductive coil measurements across a gel phantom simulation of internal bleeding in the brain. Bioelectromagnetics.

[ref-36] Oziel M, Korenstein R, Rubinsky B (2020). A brain phantom study of a noncontact single inductive coil device and the attendant algorithm for first stage diagnosis of internal bleeding in the head. Journal of Medical Devices.

[ref-37] Pan W, Yan Q, Qin M, Jin G, Sun J, Ning X, Zhuang W, Peng B, Li G (2015). Detection of cerebral hemorrhage in rabbits by time-difference magnetic inductive phase shift spectroscopy. PLOS ONE.

[ref-38] Parry-Jones AR, Wang X, Sato S, Mould WA, Vail A, Anderson CS, Hanley DF (2015). Edema extension distance: outcome measure for phase II clinical trials targeting edema after intracerebral hemorrhage. Stroke.

[ref-39] Powers WJ, Rabinstein AA, Ackerson T, Adeoye OM, Bambakidis NC, Becker K, Biller J, Brown M, Demaerschalk BM, Hoh B (2018). 2018 guidelines for the early management of patients with acute ischemic stroke: a guideline for healthcare professionals from the American Heart Association/American Stroke Association. Stroke.

[ref-40] Qusba A, Ramrakhyani AK, So JH, Hayes GJ, Dickey MD, Lazzi G (2014). On the design of microfluidic implant coil for flexible telemetry system. IEEE Sensors Journal.

[ref-41] Rao CPV, Bershad EM, Calvillo E, Maldonado N, Damani R, Mandayam S, Suarez JI (2018). Real-time noninvasive monitoring of intracranial fluid shifts during dialysis using volumetric integral phase-shift spectroscopy (VIPS): a proof-of-concept study. Neurocritical Care.

[ref-42] Rasulo FA, Bertuetti R, Robba C, Lusenti F, Cantoni A, Bernini M, Girardini A, Calza S, Piva S, Fagoni N (2017). The accuracy of transcranial Doppler in excluding intracranial hypertension following acute brain injury: a multicenter prospective pilot study. Critical care.

[ref-43] Riedel CH, Keppelen M, Nani S, Merges RD, Dössel O (2004). Planar system for magnetic induction conductivity measurement using a sensor matrix. Physiological Measurement.

[ref-44] Rosell J, Casanas R, Scharfetter H (2001). Sensitivity maps and system requirements for magnetic induction tomography using a planar gradiometer. Physiological Measurement.

[ref-45] Selim M, Norton C (2020). Perihematomal edema: implications for intracerebral hemorrhage research and therapeutic advances. Journal of Neuroscience Research.

[ref-46] Song J, Chen R, Yang L, Zhang G, Li W, Zhao Z, Xu C, Dong X, Fu F (2018). Electrical impedance changes at different phases of cerebral edema in rats with ischemic brain injury. Biomed Research International.

[ref-47] Sun J, Chen J, Li G, Xu L, Ren J, Chen M, Xu J, Bai Z, Yang J, Chen Y, Qin M, Leung K-W (2019). A Clinical research on real-time monitoring of cerebral edema after Basal Ganglia hemorrhage based on near-field coupling phase shift technology. IEEE Access.

[ref-48] Sun J, Jin G, Li G, Chen Y, Qin M (2016). The Experimental study of increased ICP on cerebral hemorrhage rabbits with magnetic induction phase shift method. 13.

[ref-49] Sun OG, Lee YJ, Koo HR, Khang S, Kim KN, Kang SJ, Lee JH, Lee J-W (2015). Application of a textile-based inductive sensor for the vital sign monitoring. Journal of Electrical Engineering & Technology.

[ref-50] Teichmann D, De Matteis D, Bartelt T, Walter M, Leonhardt S (2015). A bendable and wearable cardiorespiratory monitoring device fusing two noncontact sensor principles. IEEE Journal of Biomedical and Health Informatics.

[ref-51] Teichmann D, Foussier J, Buscher M, Walter M, Leonhardt S (2013a). Textile integration of a magnetic induction sensor for monitoring of cardiorespiratory activity.

[ref-52] Teichmann D, Kuhn A, Leonhardt S, Walter M (2013b). Human motion classification based on a textile integrated and wearable sensor array. Physiological Measurement.

[ref-53] Teichmann D, Kuhn A, Leonhardt S, Walter M (2014). The MAIN Shirt: a textile-integrated magnetic induction sensor array. Sensors.

[ref-54] Vanaclocha V, Saiz-Sapena N, Rivera-Paz M, Herrera JM, Ortiz-Criado JM, Verdu-Lopez F, Vanaclocha L (2017). Can we safely monitor posterior fossa intracranial pressure? A cadaveric study. British Journal of Neurosurgery.

[ref-55] Vinciguerra L, Bösel J (2017). Noninvasive neuromonitoring: current utility in subarachnoid hemorrhage, traumatic brain injury, and stroke. Neurocritical Care.

[ref-56] Wang J, Healey T, Barker A, Brown B, Monk C, Anumba D (2017). Magnetic induction spectroscopy (MIS)—probe design for cervical tissue measurements. Physiological Measurement.

[ref-57] Wykes V, Vindlacheruvu R (2015). Intracranial pressure, cerebral blood flow and brain oedema. Surgery.

[ref-58] Xiao Z, Tan C, Dong F (2019). 3-D hemorrhage imaging by cambered magnetic induction tomography. IEEE Transactions on Instrumentation and Measurement.

[ref-59] Xu Z, He W, He C, Zhang Z, Liu Z (2008). Measurement system of biological tissue magnetic induction tomography. 2008 world automation congress.

[ref-60] Yan QG, Jin G, Ma K, Qin MX, Zhuang W, Sun J (2017). Magnetic inductive phase shift: a new method to differentiate hemorrhagic stroke from ischemic stroke on rabbit. Biomedical Engineering Online.

[ref-61] Yang M, Wang P, Yang Z, Yuan T, Feng W (2019b). A systemic review of functional near-infrared spectroscopy for stroke: current application and future directions. Frontiers in Neurology.

[ref-62] Yang J, Zhao H, Li G, Ran Q, Chen J, Bai Z, Jin G, Sun J, Xu J, Qin M (2019a). An experimental study on the early diagnosis of traumatic brain injury in rabbits based on a noncontact and portable system. PeerJ.

[ref-63] Zakaria Z, Rahim RA, Mansor MSB, Yaacob S, Ayub NMN, Muji SZM, Rahiman MHF, Aman SMKS (2012). Advancements in transmitters and sensors for biological tissue imaging in magnetic induction tomography. Sensors.

[ref-64] Zhao S, Li G, Gu S, Ren J, Chen J, Xu L, Chen M, Yang J, Leung K, Sun J (2019). An experimental study of relationship between magnetic induction phase shift and brain parenchyma volume with brain edema in traumatic brain injury. IEEE Access.

