# Supplementary material for: A noninvasive flexible conformal sensor for accurate real-time monitoring of local cerebral edema based on electromagnetic induction
Source: PeerJ. 2020 Oct 6;8:e10079. doi: 10.7717/peerj.10079 (PMC7546241; doi:10.7717/peerj.10079)
Supplement: Table S1 [file peerj-08-10079-s001.docx]

Table 1:

Nonparametric multi-independent sample test of MIPS data as a function of bending radius at three volume

| *Volume/ml* | *Chi-Square* | *df* | *Aysmp. Sig.* |
| --- | --- | --- | --- |
| *3* | 45.07 | 3 | 8.96e^-10^ |
| *6* | 43.92 | 3 | 1.57e^-9^ |
| *9* | 50.49 | 3 | 6.28e^-11^ |
