# Supplementary material for: A noninvasive flexible conformal sensor for accurate real-time monitoring of local cerebral edema based on electromagnetic induction
Source: PeerJ. 2020 Oct 6;8:e10079. doi: 10.7717/peerj.10079 (PMC7546241; doi:10.7717/peerj.10079)
Supplement: Table S2 — Group 1/2/3/4 respectively represent flat and bending radius of 100 mm, 75 mm, 50 mm [file peerj-08-10079-s002.docx]

Table 2:

Non-parametric independent sample test of MIPS data between any pare of bending radius in each volume.

Group 1/2/3/4 respectively represent flat and bending radius of 100mm, 75mm, 50mm

| *Volume/ml* | *group* | *test Statistics* | *std. Error* | *Std. Test Statistics* | *Sig.* | *Adjusted Sig.* |
| --- | --- | --- | --- | --- | --- | --- |
| 3 | 2-3 | -3.95 | 7.16 | -0.55 | 0.58 | 1 |
|  | 2-4 | -11.47 | 7.36 | -1.56 | 0.12 | 0.71 |
|  | 2-1 | 43.25 | 7.163 | 6.04 | 0 | 0 |
|  | 3-4 | -7.52 | 7.36 | -1.02 | 0.31 | 1 |
|  | 3-1 | 39.30 | 7.16 | 5.49 | 0 | 0 |
|  | 4-1 | 31.78 | 7.36 | 4.32 | 0 | 0 |
| 6 | 2-3 | -4.43 | 7.16 | -0.62 | 0.54 | 1 |
|  | 2-4 | -9.82 | 7.36 | -1.34 | 0.18 | 1 |
|  | 2-1 | 42.70 | 7.16 | 5.96 | 0 | 0 |
|  | 3-4 | -5.40 | 7.36 | -0.73 | 0.46 | 1 |
|  | 3-1 | 38.28 | 7.16 | 5.34 | 0 | 0 |
|  | 4-1 | 32.88 | 7.36 | 4.47 | 0 | 0 |
| 9 | 2-3 | -13.35 | 7.16 | -1.84 | 0.06 | 0.37 |
|  | 2-4 | -17.63 | 7.35 | -2.40 | 0.02 | 0.10 |
|  | 2-1 | 49.08 | 7.16 | 6.85 | 0 | 0 |
|  | 3-4 | -4.28 | 7.36 | -0.58 | 0.56 | 1 |
|  | 3-1 | 35.73 | 7.16 | 4.99 | 0 | 0 |
|  | 4-1 | 31.44 | 7.36 | 4.27 | 0 | 0 |
